# Alleviation of Asthma Symptoms After Ketogenic Diet: A Case Report

**DOI:** 10.7759/cureus.34526

**Published:** 2023-02-01

**Authors:** Muath Al-Rebdi, Unaib Rabbani

**Affiliations:** 1 Family Medicine, Qassim Health Cluster, Buraidah, SAU

**Keywords:** management, ketogenic diet, control, case report, asthma

## Abstract

Asthma is a chronic disease that affects the quality of life of patients, and asthma exacerbations are often a reason for hospitalization and activity limitations. Obesity has been linked to asthma as a risk and exacerbating factor. Evidence suggests that weight reduction has a positive effect on asthma control. However, there is also debate on the role of the ketogenic diet in asthma control. Here we present a case of asthma who reported markedly improved asthma after starting a ketogenic diet in the absence of any other lifestyle change. Over the four months on the ketogenic diet, the patient reported losing 20 kg of weight, reduction in blood pressure (without antihypertensives), and complete alleviation of asthma symptoms. This case report is important as the control of asthma after a ketogenic diet is not studied well in humans and therefore needs to be studied extensively.

## Introduction

One common diet regimen nowadays which showed rapid weight loss is the ketogenic diet (KD). This type of diet primarily consists of high fats, moderate proteins, and very low carbohydrates (less than 10%) [1]. Historically, KD has been used in pediatric epilepsy [2]. The significant benefit of KD is reported in a meta-analysis of randomized controlled trials by Choi et al., which found improvement in metabolic parameters such as glycemic, lipid, and weight [3]. Regarding the safety of the ketogenic diet, Dashti et al. concluded that KD was effective in reducing body weight and safe in the long term [4]. The biochemistry process behind the ketogenic diet is ketogenesis in which there is the production of ketone bodies as an alternative source of energy to glucose.

Many investigators suggest that obesity plays role in asthma [5]. It is a risk factor as well as an exacerbating factor. A meta-analysis of prospective studies found a positive association between weight loss and improving asthma attacks [6]. Many researchers question whether the improvement of asthma among obese on a ketogenic diet is because of weight loss or the nature of the diet. Evidence exists on the positive effect of weight loss on asthma attacks [7]. However, an animal study showed that dietary ketone increased body β hydroxybutyrate (BHB) concentration and decreased methacholine hyperresponsiveness in mice [8], consequently, relieving asthma symptoms without the need for weight loss [8].

During a routine follow-up at a primary health care center, an asthmatic patient drew our attention to a report of asthma control following a ketogenic diet. We present here the details of the case.

Informed consent was obtained from the patient for reporting his case without any personal information. Approval to publish the case report was obtained from Qassim Regional Bioethics Committee (Approval number: 607-44-2086).

## Case presentation

A 32-year-old Saudi national, who is an English teacher by profession and married with two children, was known to have obesity and bronchial asthma (BA) since childhood. He came to the family medicine clinic for regular BA follow-up and refill inhalers on 13th March 2022.

Asthma medications he was taking included a combination of fluticasone propionate and salmeterol 125 bid 2 puffs, and salbutamol as needed. He had a positive family history of bronchial asthma as his grandfather had died because of an asthma attack. Shortness of breath, cough, and audible wheezing chest were usual symptoms during his asthma exacerbations. His asthma triggers were seasons transitions, exercise, animals like horses, and some fruit like melons. Regarding the level of control, according to Asthma Symptom Control, which is adapted from the Global Initiative for Asthma (GINA) guidelines [9], he reported one night attack per month, which sometimes affected his sleep. He used salbutamol to relieve the symptoms. He also mentioned a sport limitation as a result of asthma. On examination, his weight was 107 kg, height 175 cm, and BMI 34.9, while BP was 155/92 mmHg (the reading of BP on the last visit was 145/93 mmHg on 27th February 2022). Thus, our assessment was uncontrolled asthma symptoms and primary hypertension. Based on these, the dose of fluticasone propionate and salmeterol combination was increased to 250 bid 2 puff, and valsartan 80 mg antihypertensive medication was included in the prescription.

On the 17th July 2022 visit, he reported that he has started a new type of diet which is called a ketogenic diet to lose weight since 18th of March 2022. He reported losing more than 20 kg and complete alleviation of asthma symptoms. Since starting the keto diet, he denied any asthma exacerbations or sport limitation and he stopped using his inhalers. He also reported no other change in his lifestyle. When we used Asthma Symptom Control to determine the level of control, he has imporved and asthma symptoms were controlled (Table 1). He also reported not initiating antihypertensive medication. The BMI on July visit was 28 and BP was 130/82 mmHg.

**Table 1:** GINA assessment of asthma control of the case before and after the start of the ketogenic diet *Based on short-acting beta_2_-agonist (SABA) and excludes reliever taken before exercise. GINA: Global Initiative for Asthma

Asthma symptoms control	Before	After
Daytime asthma symptoms more than twice/week?	Yes	No
Any night waking due to asthma?	Yes	No
Reliever needed for symptoms more than twice/week?*	Yes	No
Any activity limitation due to asthma?	Yes	No
Level of Asthma Symptom Control	Uncontrolled	Controlled

## Discussion

Asthma is a chronic disease that is associated with high morbidity and mortality globally. Asthma control is the main strategy to improve the functionality and quality of life of patients. This requires various medications and prevention of exposure to asthma triggers. Obesity has been linked to increased asthma exacerbations, and weight control has been linked to reduced severity of asthma. In this case report, we observed that after initiating a ketogenic diet, there was an improvement in the symptoms of an asthmatic patient.

As we go back to the case, we found that the patient had a gradual improvement in asthma symptoms despite not taking his medications. As the patient mentioned that there was no change in extrinsic factors, we searched to find out if intrinsic factors could be the cause. Of course, weight reduction played the main role in the improvement of lung functioning because of decreased soft tissue that compresses the thoracic cage, decreases fat infiltration, and decreases pulmonary blood volume. It is suggested that a systemic proinflammatory process induced by adipose tissue could be playing role in an asthmatic obese patient [10]. However, previous studies have shown that there was no link between obesity and objective markers of asthma and airway obstruction [11]. Another study also finds better lung function in obese asthmatics as compared to lean asthmatics [12]. Recent evidence, on the other hand, shows a strong role of inflammatory processes in asthmatics, triggered by obesity [13]. 

Food is considered one of the triggers of asthma symptoms. Buendía et al. found an association between high carbohydrate-rich food intake and asthma severity in children [14]. Mank et al. concluded that dietary ketone relieves asthma symptoms without the need for weight loss in mice [8]. Lifestyle changes and weight reduction can lead to a reduction in blood pressure even without the initiation of medication [15]. Further evidence on the role of diet in asthma control shows that the effects of specific dietary factors are equally important as excessive weight in the pathophysiology of asthma [16]. It is also suggested that improving diet quality can be effective in the control of asthma symptoms [16].

## Conclusions

Based on available evidence it can be suggested that, along with a reduction in weight, the ketogenic diet might have also played an independent role in the alleviation of asthma symptoms of our case. In conclusion, both weight reduction as a primary factor that was approved by studies, and the ketogenic diet as a secondary factor that needs more investigation, have significant positive effects on asthma improvement. In this regard, further prospective studies or randomized controlled trials should be carried out to establish the effectiveness of KD in the control of asthma.
